# From Environment to Landscape. Reconstructing Environment Perception Using Numerical Data

**DOI:** 10.1007/s10816-015-9264-9

**Published:** 2015-10-26

**Authors:** Cătălin Nicolae Popa, Daniel Knitter

**Affiliations:** 1 Excellence Cluster Topoi, Freie Universität Berlin, Topoi Building Dahlem, Hittorfstraße 18, D-14195 Berlin, Germany; 2 Excellence Cluster Topoi, Department of Earth Sciences, Institute of Geographical Sciences, Physical Geography, Freie Universität Berlin, Malteserstraße 74-100, D-12249 Berlin, Germany

**Keywords:** Landscape, Environment, Perception, Fuzzy logic, Statistical modelling

## Abstract

The paper introduces a method that links environment to landscape. The environment-landscape divide appears because of epistemological differences: since studying the landscape involves describing the world as it was perceived by humans, it is difficult to access this dimension through the numerical data that we employ when studying the environment. We approach the issue of non-correspondence between environment and landscape knowledge using fuzzy logic. The numerical data describing two geomorphometric parameters, slope and modified topographic index, are split each into three classes with overlapping borders. The classes are then fused into four qualitative categories: flat wet, steep dry, flat dry, and gradual moist. These four categories have direct correspondence in the real world and can be observed by people through simple perception. The correspondence of such categories to peoples’ perception is checked against evidence of past human settlement in three areas coming from Turkey, Serbia, and Syria. The identified qualitative categories resemble the way people categorized their landscape in all but the second case study. Humans were able to perceive and choose areas which correspond to gradual moist in Turkey and broadly to flat wet in Syria. However, for the Serbian example, the results are inconclusive.

## Introduction

We may never fully understand how prehistoric people perceived their surroundings, but such knowledge is not entirely out of our reach. The main difficulty that scholars encounter stems from the division between environment and landscape. This separation, already signalled by authors such as Ingold (2000, pp. 209–218) and Meier (2012), is based on the different role that humans play within such studies. Meier argues that environment-focussed studies are concerned with the world in relation to which humans are external observers, while landscape-orientated approaches place people at their centre. Despite the widespread use of the word landscape, most studies actually focus on the environment because they concentrate on quantifying its different aspects. In contrast, landscape is the outcome of people’s perception and engagement with the world around them (Bender 1993, p. 1), and as a result, its characteristics are far less quantifiable. In this paper, we introduce a method that links environment to landscape by employing modern measurements to deduce the way in which past people might have perceived their surroundings. The method does not aim to provide clear answers or produce predictions but rather to offer a more rigorous approach to our understanding and interpretation of the relationship between people and landscape.

The topic of perception has been tackled in archaeology in relation to landscape mainly from two directions. On one hand, phenomenological approaches, based primarily on the works of Heidegger (1993, pp. 343–364) and Merleau-Ponty (1962), have sought to produce an embodied experience of the landscape in which perception, together with bodily actions, movements and emotions, play a fundamental role (Thomas 1993, 1996; Tilley 1994). Although attractive in terms of their scope, phenomenology-centred studies have been criticized for their limited scale and lack of formal methods to substantiate their theoretical ideas (e.g. Tilley 1996; Watson 2001; for a critique, see Fleming 1999, 2006; Brück 2005). On the other hand, perception has occasionally been incorporated into Geographic Information Systems (GIS). In such GIS studies, the existence of a perceived environment is acknowledged; such perception is reflected by the real environment (Butzer 1982, pp. 252–259; Sonnenfeld 1972) and is approached through the notion of affordances, seen as the specific combination of properties, substance and surface, with reference to an animal, object or place (Gibson 1977). Affordance-based GIS methods have found only limited implementation, principally in the works of Llobera (1996, 2001, 2012) and Gillings (2007, 2009, 2012). While showing great promise, they have been criticized for maintaining an objectivist, Cartesian model of space (Brück 2005, pp. 52–54; Thomas 2004, pp. 198–201). In fairness, this criticism can be extended to all GIS-based studies.

Our paper explores perception as a means to connect environment and landscape, using Meier’s (2012) definition of the two terms, without resorting to the notion of affordances. Instead, perception is approached starting from people’s sensorial and cognitive abilities.

### From Environment to Landscape

Since studying the landscape involves describing the world as it was perceived by humans, it is difficult to access this dimension through the data and measurement tools that we employ for the environment. Furthermore, there is the additional issue of the precision of modern measurements. Thanks to modern devices, we can obtain detailed information about the environment (e.g. amount of rainfall, variation in surface topography, etc.), whereas past humans did not have such instruments. People employed their senses when interacting with the world around them and took decisions based on this information, among other factors. Therefore, if we seek to study the landscape, we need to understand how people might have experienced their surroundings using their senses alone, by interpreting precise data to get a measure of this process.

A further issue, representing a corollary of the points above, rises from the contrast between the numerical character of our data and the categorical nature of people’s perception. The methods deployed when studying the environment provide us with precise, numerical, continuous data, but the information that humans employ in their daily lives is mainly categorical and inexact in nature. Cognitive psychologists explain this categorization of our world as a process that is essential to human brain mechanics; humans understand reality by segmenting it into categories, each of which is characterized according to a specific set of properties (Boyer 2010; Boyer and Ramble 2001; Kurzban *et al.* 2001). It is possible that the importance of categorization as a cognitive process is higher today than in prehistoric times (McGilchrist 2010), but there is little doubt among psychologists and neuroscientists that categorization was and remains a fundamental brain mechanism that shapes our understanding of the world (Burleigh and Schoenherr 2015; Lamberts and Shanks 1997; van Mechelen *et al.* 1993; Rosch 1978). The employed categories come out of perception, being informed by the senses, but also by past experiences and cultural norms. They often have an overarching character, grouping together different elements, and have a large degree of inexactness and variation, making it hard to draw exact boundaries around them. This kind of world construction is in direct opposition to the way in which we tend to gather data in our discipline, using devices that measure individual elements of the environment and produce precise numerical values. In short, this contrast entails fundamental epistemological differences.

We approach this issue of non-correspondence between environment and landscape knowledge using fuzzy logic. The numerical data describing two environment elements are split into fuzzy classes using a series of empirically derived and contextually adapted intervals. The fuzzy classes are then merged into combination sets and further fused into qualitative categories. The reliance on fuzzy classes allows for the information recorded using modern measurement tools to be connected with the observational capacities of the senses. In addition, the combination of fuzzy classes from different variables enables us to get closer to the categories employed by people when acting within the landscape, since these were not based on a single element but were rather more inclusive. The correspondence of the qualitative categories to people’s perception of the world, and thus to landscape, is checked against the archaeological evidence. The input for the analysis consists of two geomorphometric parameters derived from digital elevation models, which comprehensively express observable qualitative differences: slope and modified topographic index (MTI).

### Fuzzy Logic

Fuzzy logic is a concept introduced by Zadeh (1965) that helps to define classification systems in instances where clear borders are hard to establish. This can prove useful since many concepts that humans employ in daily life are of a relative nature. The notions of hot and cold are an example of this. While most people use the two terms to characterize aspects of the world around them, it is hard and arguably inappropriate to draw a clear border between the two. Fuzzy logic allows for such uncertainties to be incorporated in a classification system.

The main idea behind fuzzy logic is, rather than to assign each case to a particular category, they are all assigned a membership value for all possible fuzzy classes. This membership value is expressed as a figure between 0 and 1 and signifies the degree to which that particular case is included in one of the possible fuzzy classes. Therefore, each case will be part of all classes, but it will have a stronger membership value for some than for others. For instance, water at 10 °C is both hot and cold, but it is in many contexts more cold than hot.

Despite the diversity they can accommodate, fuzzy classes should be defined contextually rather than universally. The categories that people employ when referring to particular elements of their world can be different from one context to another and the borders drawn between them can undergo considerable alterations (e.g. 10 °C water may be considered hot in some contexts and cold in others). While fuzzy classes can accommodate some degree of shifting borders, if the classes are too wide, they can become meaningless for explaining human behaviour in particular situations. Consequently, fuzzy classes should be defined based on the information available in the context that is being studied, since that is also the information that would have been available to past humans.^1^ However, in some situations, more general, empirically derived classes can be used.^2^

Although resorting to fuzzy logic in archaeology is not a novelty, its implementation remains limited. Some have argued for its use when developing artefact typologies, since past people likely employed general object categories rather than made distinctions based on precise measurable differences (Hermon and Nicculucci 2002, 2003; Hermon *et al.* 2004). In another instance, fuzzy logic was combined with agent-based modelling to describe patterns of agricultural use in Iron Age Europe (Machálek *et al.* 2013). Zadeh’s ideas have also been employed when making chronology inferences (Nakoinz 2012) or in combination with correspondence and cluster analysis (Baxter 2009; Riedhammer 1997). Occasionally, fuzzy logic has been integrated into GIS models, particularly in connection with viewshed methods (Loots *et al.* 1999; Rášová 2014) but also for the general analysis of spatial data (Jarosław and Hildebrandt-Radke 2009; Jasiewicz 2011). Overall, although far from extensive, the applications of fuzzy logic in archaeology are encouraging, showing its potential for understanding how humans interacted with the world around them.

## Method Description

### Geomorphology

Our main data are Digital Elevation Models (DEM) of today’s topography. Topography describes the general configuration of the land surface, defined by latitude, longitude and altitude. It “(…) can act directly on [local] climate, water flow and storage, soils and sediments and living things” (Huggett 2010, p. 170). Topography also steers geomorphological processes, like weathering or erosion, though their specific pattern is related to geological and climatic characteristics. Consequently, topography represents a common denominator for studies focusing on questions where location is important.

Topography has great utility for studying the past, because it has a slow pace of change. While some of the earth’s other spheres, such as the atmosphere and biosphere, are characterized by fast transformations, the topography, as part of the geosphere, is less sensitive. In general, the larger the extent of specific topographic features or geomorphological forms, the longer their existence (Ahnert 1981). Following this empirical rule, the overall surface characteristics of an area are relatively constant across millennia when approached on a regional scale (Ahnert 1981, p. 9). Therefore, given its slow changing nature, the general past topographic conditions can be described using the modern day DEM. From the DEM, we extract two geomorphometric parameters.

Our first parameter is slope, a primary land-surface attribute (Wilson and Gallant 2000, p. 1ff) that describes the magnitude of gradient in the rate of change of the elevation on the *x* and *y* axes, calculated using a neighbourhood matrix (e.g. Albrecht 2007, p. 62; Olaya 2009, p. 144). Slope is fundamental for a series of processes, such as the velocity of surface and subsurface flow, soil water content, erosion potential, soil formation, etc. (Gallant and Wilson 2000, p. 53). We calculate the slope in degrees using the GRASS GIS software and the r.param.scale package (Hofierka *et al.* 2009), which fits a bivariate quadratic polynomial to a given window size, in our case 3 × 3 pixel, using least squares (Wood 1996, p. 84).

Since slope magnitude strongly influences soil and water movements, and thus erosion, it represents a limiting factor for environment utilization. Results of empirical analyses allow us to classify slope in classes that link its value to potential human practices. Below 5 % (2.86°) slope, areas are suitable for agricultural purposes and erosion is a minor problem. Pixels corresponding to this characteristic belong to the fuzzy class small. The critical slope for construction is 8 % (4.57°), since at this point, erosion increases significantly. The fuzzy class moderate covers pixels up to this threshold. The fuzzy class high refers to slope values higher than 8 % and particularly above 10 % (5.7°), when erosion becomes a severe problem and land utilization is only possible with large efforts (empirical values based on Cooke and Doornkamp 1974, p. 361; Leser 1968, p. 38; Pécsi 1985).

The second parameter corresponds to a second-order surface attribute and represents a modified version of the topographic index. Such secondary attributes serve to describe the surface as a function of real processes (Wilson and Gallant 2000, p. 6). The topographic index quantifies the influence of topography on the redistribution of water throughout an area. For instance, the topographic characteristics determine the distribution and availability of soil water and accordingly the distribution and abundance of flora and fauna as well as the susceptibility to erosion by water. The topographic index quantifies this topography-induced influence on the hydrological, geomorphological and ecological characteristics of an area (Beven and Kirkby 1979, pp. 44–45; Wilson and Gallant 2000, p. 6). The topographic index is defined as

$$ TI= ln\left(\frac{A}{tan\beta}\right) $$

where *A* is the specific catchment area, calculated using r.watershed in GRASS GIS, and *tanβ* is the tangent of slope gradient at that location (e.g. Moore *et al.* 1991, p. 13). In order to get more specific information on the flood susceptibility of an area, we use a modified version of the topographic index (MTI) developed by Manfreda *et al.* (2011). In the MTI, the relative weight of the drained area (i.e. the specific catchment area) is altered by introducing an exponent (*n*). The modified topographic index takes then the form

$$ MTI= ln\left(\frac{A^n}{tan\beta}\right) $$

The MTI is not only a good indicator of areas exposed to floods but also of parts of the topography with little and intermediate amounts of converging water. High MTI values signal topographical locations with large amounts of water; conversely, small MTI implies a topographically induced scarcity of water. Intermediate values of the MTI correspond to areas of moderate water concentration, which occurs on footslopes and alluvial fans. These three possible scenarios are represented through the fuzzy classes small, moderate and high. However, unlike slope, there are no empirical thresholds for the three fuzzy classes since the MTI is calculated using the specific catchment of each region. Therefore, the limits between small, moderate and high MTI can vary from case to case.

**Fig. 1:**
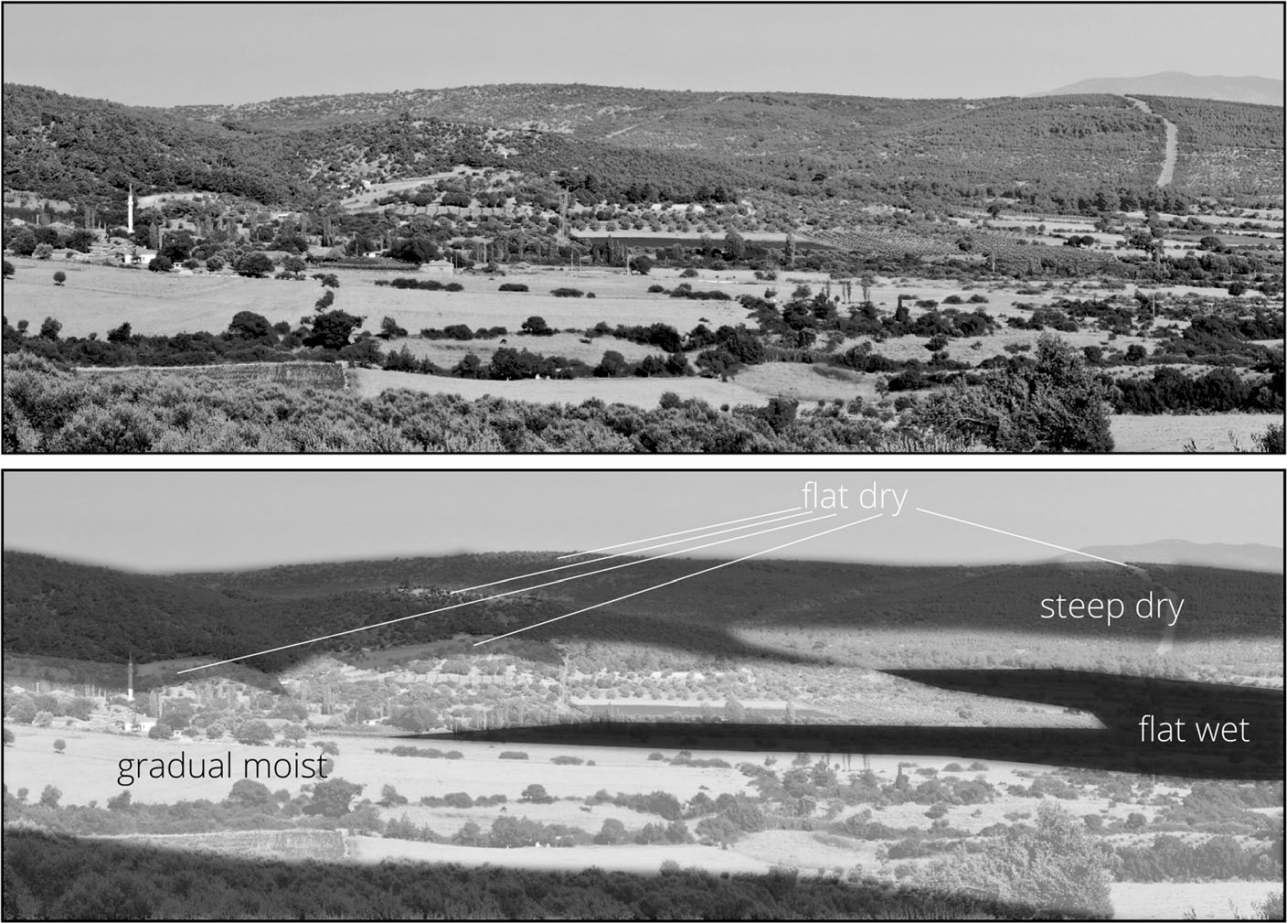
*Top*: example of environment from Western Turkey. *Bottom*: distribution of the four qualitative categories in the exemplified environment

Combined slope and (modified) topographic index are the terrain attributes that correlate best with vegetation, particularly when taken together with climate and with surface soil attributes (Moore *et al.* 1993), such as nutrient availability and distribution. Consequently, we employ slope and MTI fuzzy classes to categorize the environment into four areas with similar characteristics: flat wet, steep dry, flat dry and gradual moist (Table 1, Fig. 1). These four qualitative categories have direct correspondence in the real world and can be observed by people through simple perception. They distinguish real-world environments in categories that are different in terms of topography and accordingly in terms of vegetation composition and soil characteristics. This leads us to the hypothesis that they offer a good description not only of the environment but also of the landscape, since humans probably perceived them as different. However, this hypothesis needs to be contextually confirmed by comparing the distribution of the qualitative categories with the patterns of human activity. A more detailed classification is possible but not reasonable, since this calls for (a) more input parameters, which complicates the basic methodology, (b) higher resolution of the input data, which is not available everywhere and (c) it would give excess weight to the specific, large-scale characteristics of the present surface conditions, which is misleading in terms of the evolution and sustainability of geomorphological forms (see Ahnert 1981).

**Table 1:** Description of the four qualitative categories

Name	Description	Fuzzy set correspondence		Real-world correspondence
		Slope	MTI	
Flat wet	Flat areas liable to water concentration	Small	High	Floodplains; wetlands
Steep dry	Steep areas with little to no water concentration	Moderate or high	Small	Mountainous areas
Flat dry	Flat areas with little to no water concentration	Small	Small	Watershed divides; small plateaus along extensive slopes
Gradual moist	Flat to gently inclined areas of intermediate water concentration	Small or moderate	Moderate	Footslopes; alluvial fans

### Fuzzification Procedure

The fuzzification procedure involves the transformation of the exact, continuous values of the slope and MTI into membership degrees to the three fuzzy classes: small, moderate and high. The calculation takes as input four parameters for slope and another four for MTI.^3^ The two groups of four parameters are analogous and employed in a similar manner. They refer to the absolute boundaries between the fuzzy classes and define the fuzzy ranges (Table 2).

**Table 2:** Fuzzification parameters

Symbol	Parameter type	Fuzzy sets affected	
a	Absolute boundary	Small	Moderate
b	Fuzzy range	Small	Moderate
c	Absolute boundary	Moderate	High
d	Fuzzy range	Moderate	High

The membership degree for the three fuzzy classes used to split slope and MTI is calculated in the following manner:

- Fuzzy class small:
  $$ {m}_s=\left\{\begin{array}{c}\hfill 1,x\le a-b\hfill \\ {}\hfill E{Q}_s(x),a-b<x<a+b\hfill \\ {}\hfill 0,x\ge a+b\hfill \end{array}\right. $$
- Fuzzy class moderate:
  $$ {m}_m=\left\{\begin{array}{c}\hfill 1,a+b\le x\le c-d\hfill \\ {}\hfill E{Q}_m(x),a-b<x<a+bVc-d<x<c+d\hfill \\ {}\hfill 0,x\le a-bVx\ge c+d\hfill \end{array}\right. $$
- Fuzzy class high:
  $$ {m}_h=\left\{\begin{array}{c}\hfill 1,x\ge c+d\hfill \\ {}\hfill E{Q}_h(x),c-d<x<c+d\hfill \\ {}\hfill 0,x\ge c-d\hfill \end{array}\right. $$

where *x* represents the continuous value (for slope or MTI) and *EQ*_*s*_, *EQ*_*m*_ and *EQ*_*h*_ represent the equations used to calculate the membership degree for values in fuzzy intervals for fuzzy class small, moderate and high, respectively. *m*_*s*_, *m*_*m*_ and *m*_*h*_ represent the calculated membership degree at point *x* for fuzzy class small, moderate and high, respectively.

**Fig. 2:**
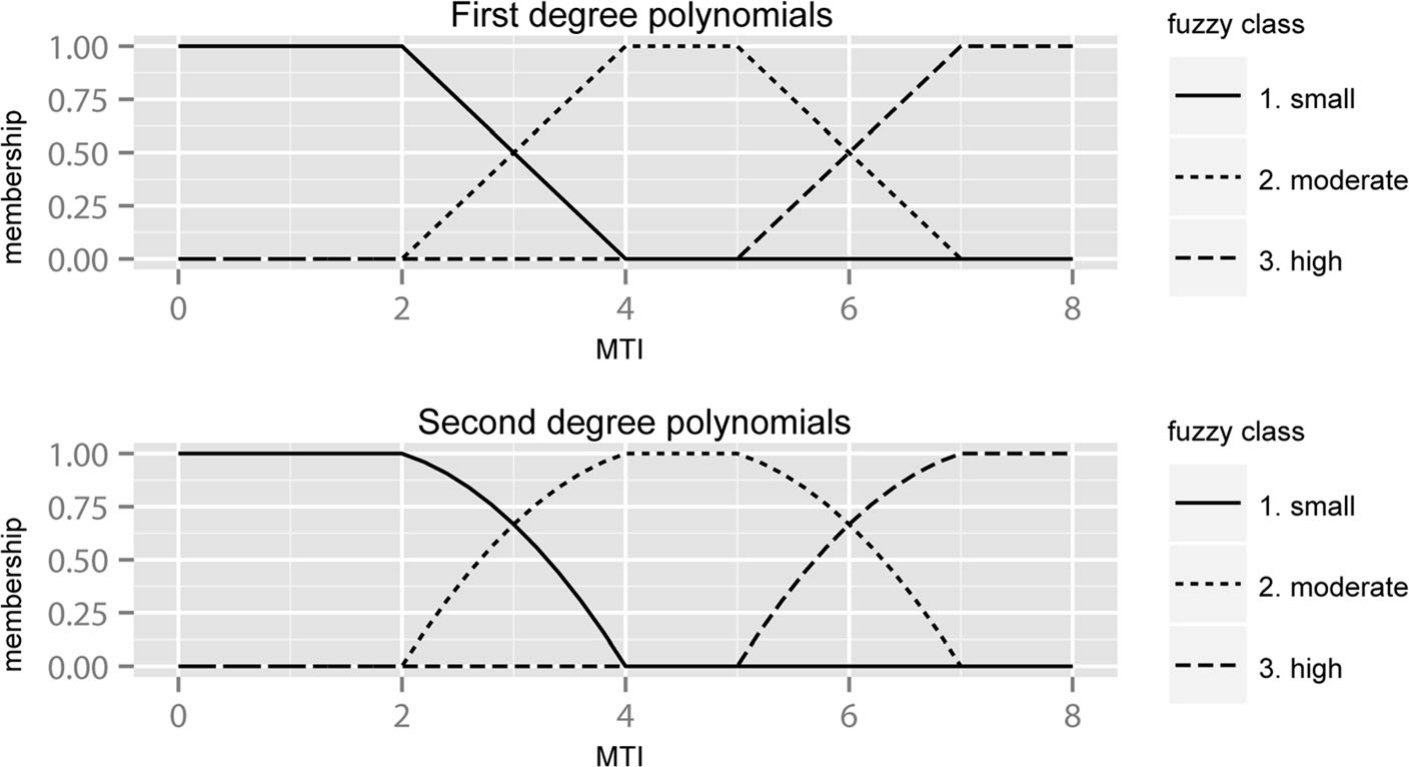
Graphical distribution of membership degree for MTI fuzzy classes

The membership degree for the values situated within fuzzy intervals is calculated with three simple polynomial equations, using the parameters described in Table 2 to determine their coefficients. The polynomials can be opted to be of either the first or the second degree, producing different fall-off curves (Fig. 2). This was not found to have a large impact on the output, though the second-degree polynomial equations generally produced more accurate results.

### Defuzzification Procedure

The defuzzification procedure refers to the combination of the slope and MTI fuzzy classes and their translation into the qualitative categories which may have been used by humans in their decision-making process. This procedure involves firstly the computation of combination sets by connecting all slope and MTI fuzzy classes to each other. Chosen combination sets are then merged further to obtain the membership values to the four qualitative categories. The maximum of these values gives the final, crisp qualitative category.

By grouping each slope fuzzy class with every MTI fuzzy class, a maximum of nine combination sets are obtained, but only some correspond to qualitative categories. Three of these combination sets cannot occur in real-world conditions (moderate slope with high MTI, high slope with moderate MTI and high slope with high MTI) and are thus discarded. Two of the remaining combination sets: small slope with small MTI and small slope with high MTI, already define qualitative categories, namely flat dry and flat wet. The final four combination sets are further combined two by two to produce the remaining two qualitative categories: gradual moist and steep dry.

**Fig. 3:**
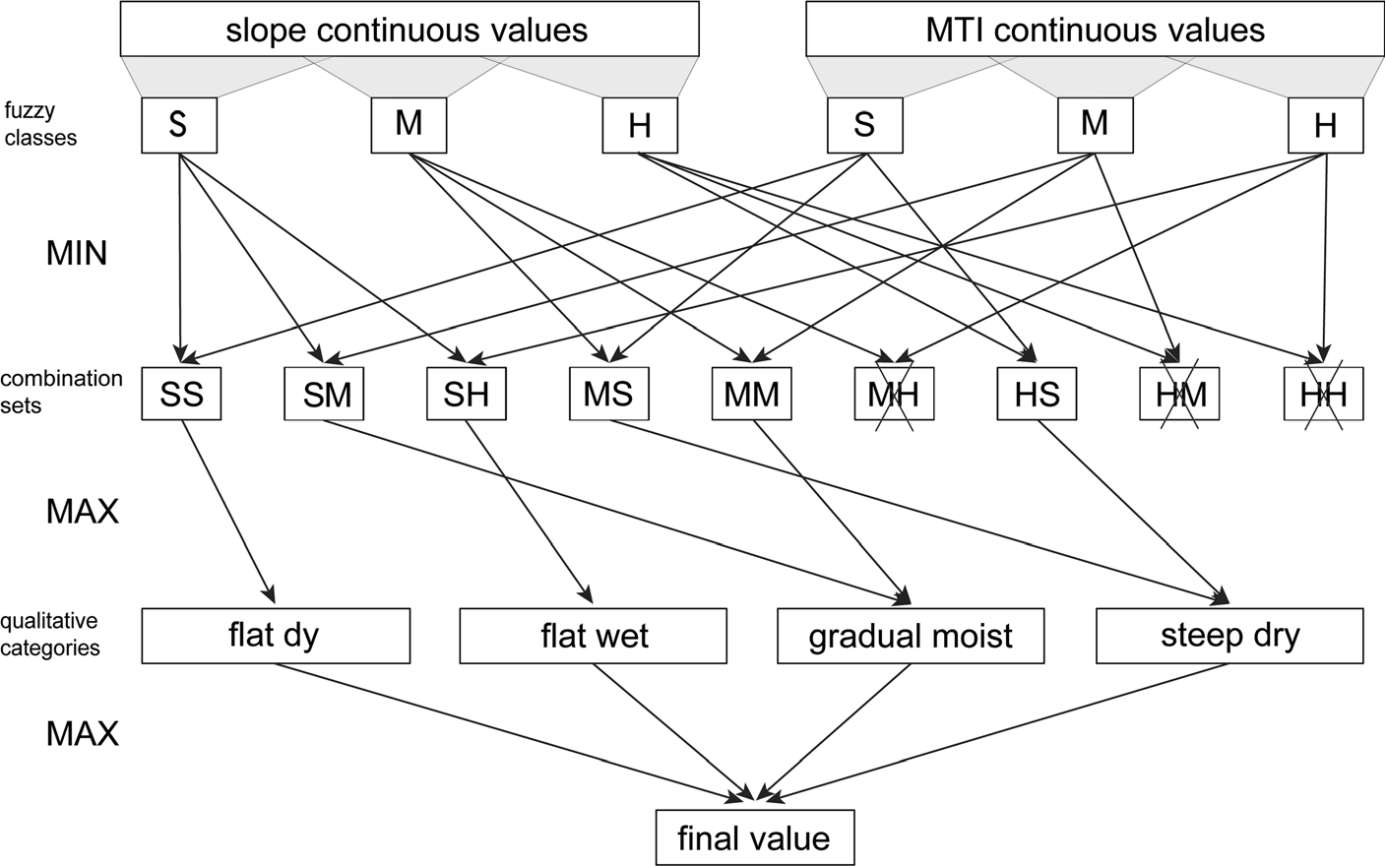
Overview of defuzzification procedure. Crossed out combination sets do not exist in real-world conditions and are thus not considered

Merging of combination sets is done using the method proposed by Zadeh (1965), which involves choosing the highest minimum values from the membership degree of the slope and MTI fuzzy classes. The first step is to produce all possible combination sets by selecting the minimum membership degree between the combined slope and MTI fuzzy classes (e.g. the minimum value between small slope and small MTI and separately the minimum value between moderate slope and small MTI). The second step is to select the maximum value for the merged combination sets, which represents the membership degree to the resulting qualitative category (Fig. 3). This procedure is repeated for each of the two qualitative categories that require the merging of combination sets.

In the last step, each pixel is assigned to a crisp qualitative category in order to produce an output map. In every case, the qualitative category with the highest membership value is chosen. However, the fuzzy membership of each qualitative category is also retained and employed in the interpretation.

### Correlation to Settlement Location

The resulting map is checked against the distribution of past settlements in order to observe whether there is a preference towards one or more of the qualitative categories. This involves calculating the percentage of the surface area occupied by each qualitative category within the convex hull^4^ defined by the settlements (Baddeley and Turner 2005; Krivoruchko and Bivand 2008). Afterwards, the percentage of settlements situated within the four qualitative categories is computed. The first of these values gives a measure of the precision of the results, while the second informs us about their accuracy (Verhagen 2007, pp. 93–94). The combination between accuracy and precision serves to calculate the gain, which reveals whether the results have predictive qualities (Kvamme 1988; Mink *et al.* 2006). This last step represents an extension of the method, since producing predictions is not our goal.

For this last step, we assume that the settlement sample is representative for the investigated areas and that their signalled locations are similar to those in the past. However, since we are dealing with archaeological data and much of the settlement information is often gathered through field surveys, there is no guarantee that our two assumptions are fully correct. It is likely that the samples are affected to some degree by surface processes: Erosion might relocate archaeological material, while sedimentation might cover settlement evidence. Such data biases can be addressed through more detailed geomorphological studies, but this information is rarely available, particularly when working with relatively large areas. This was unfortunately also the situation in our three case studies.

### Case Studies

**Fig. 4:**
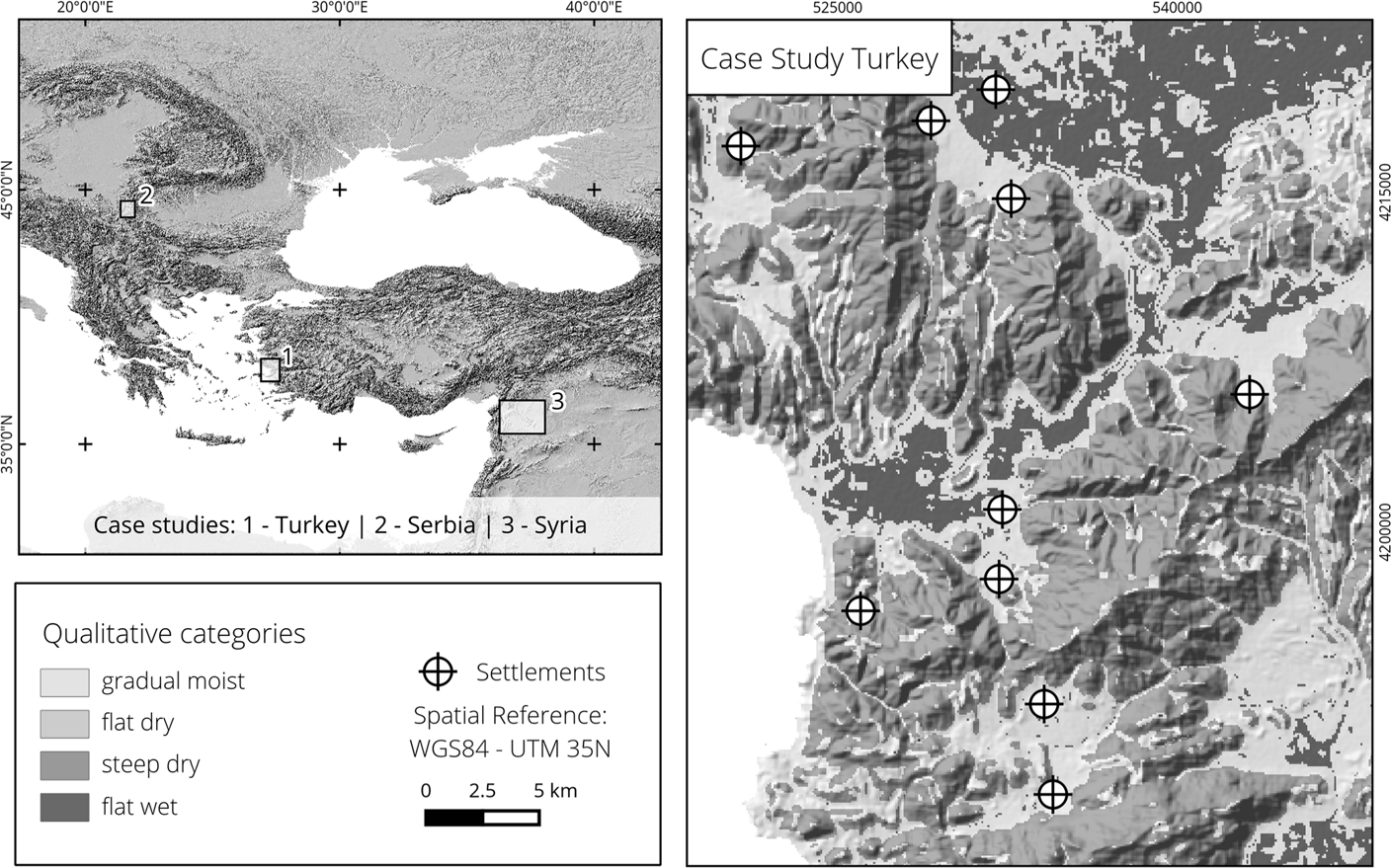
*Left*: overview map showing the location and size of the three research areas. *Right*: study area in Western Turkey (settlements from Horejs 2011; elevation data from Jarvis *et al.* 2008)

We selected three case studies located in modern day Turkey, Serbia and Syria (Fig. 4, left). An important reason behind this choice is of course linked to data availability. Additionally, we opted to include regions where the method performs well and cases where the outcome is problematic.

The first case study is represented by an area from Western Turkey, in the surroundings of ancient Ephesos. The area is presently characterized by a temperate climate (Cs after Köppen and Geiger) with humid and temperate winters and hot, dry summers (Kottek *et al.* 2006, p. 261). The topography of Western Turkey is mainly the result of geologically young (i.e. Neogene) structural movements that caused the development of mountain and graben systems, stretching from east to west (Hütteroth and Höhfeld 2002, pp. 37–39). Alluvial sediments fill up the grabens, which are drained by large river systems such as the Küçük Menderes. The large rivers of Western Turkey actively prograde their deltas into the Aegean Sea owing to high sediment load (Brückner 2005; Brückner *et al.* 2005, 2006). Furthermore, the strong, seasonal river regime, with low flow during summer and autumn and high flow during winter and spring, makes the rivers impossible to navigate (Hütteroth and Höhfeld 2002, pp. 91–92). The predominant soils are Cambisols and Luvisols. Fluvisols are present in the alluvial plains, while Leptosols are characteristic for the mountainous areas (Erol 1983, pp. 84–85; European Soil Bureau Network 2005, p. 87, Plate 16; Walter and Breckle 1991, pp. 12–14)

For our analysis, we use the DEM based on Shuttle Radar Topographic Mission (SRTM) data provided by Jarvis *et al.* (2008) with a resolution of 90 by 90 m. The plotted settlements, which amount to only ten sites, date to the Bronze Age and were collected by Horejs (2011).

**Fig. 5:**
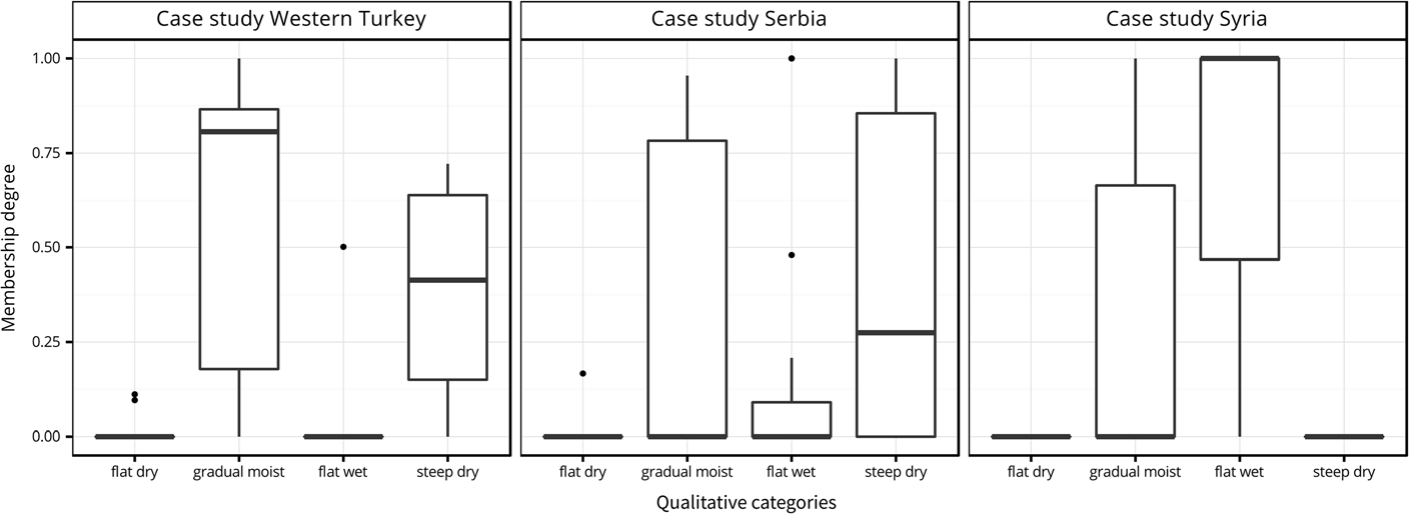
Box plot graphs with the membership degrees to the four quantitative categories at the location of the settlements, for each of the three case studies

**Fig. 6:**
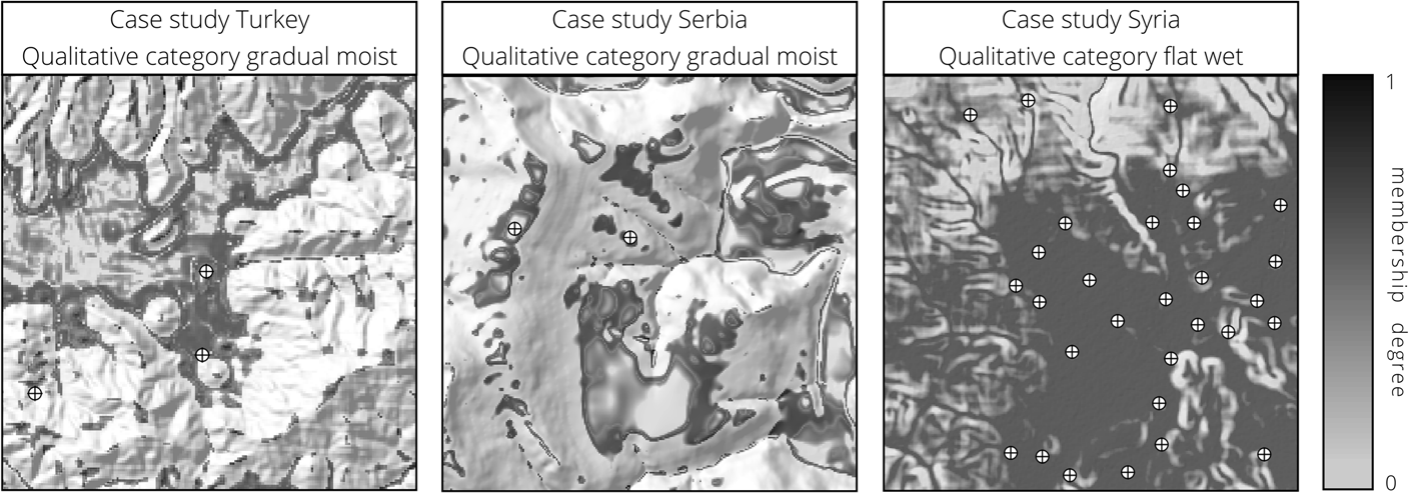
Details of the three study areas. The membership degree to a chosen qualitative category is represented using a grey colour ramp

The results of our analysis show three distinct zones (Fig. 4, right). Firstly, the mountainous areas are included in the category steep dry, which make up the largest parts of the study area. Secondly, the basins, floodplains and valleys categorized as flat wet and covering around 9 % of the surface. Finally, the footslopes, alluvial fans and terraces are categorized as gradual moist and span over one quarter of the study area. The category flat dry is missing. The analysis of the settlement locations reveals that they are heavily concentrated in the gradual moist parts (Table 3). The distribution of the fuzzy membership degrees for the four categories also shows a preference for areas that are gradual moist, as the site locations gave a median value of over 0.75 for this qualitative category (Fig. 5, left), although the graph has to be read with caution given the small number of sites. A detailed look at the gradual moist fuzzy membership shows a clear border between footslope situations and mountains, while the transition to the floodplain is gentle and displays undulating values (Fig. 6, left). A small number of sites are found in steep dry areas but never in flat wet environments.

**Table 3:** Qualitative category distribution, site distribution and gain for the three case studies

Turkey	Gradual moist	Flat dry	Steep dry	Flat wet
Area (%)	25.8	0	65.2	8.99
Sites (%)	70.0	0	30.0	0
Gain	0.63	NaN	−1.17	Inf
Serbia	Gradual moist	Flat dry	Steep dry	Flat wet
Area (%)	24.6	0.07	65.8	9.46
Sites (%)	36.0	0	52.0	12.0
Gain	0.31	Inf	−0.26	0.21
Syria	Gradual moist	Flat dry	Steep dry	Flat wet
Area (%)	50.6	0	2.65	46.8
Sites (%)	30.8	0	0	69.2
Gain	−0.64	NaN	Inf	0.32

In our second case study, we analyse data from the surroundings of the imperial palace of Felix Romuliana in Eastern Serbia. The area is characterized by heterogeneous relief with a complex geologic history. Various rock types have caused the occurrence of diverse ores and numerous geomorphological features (Bogdanović 1977). According to Jović (1997), these characteristics have favoured the early and prolonged settling of the area by humans. At present, a temperate climate prevails (Cfa after Köppen and Geiger) with dry, hot summers and temperatures of over 22 °C (Peel *et al.* 2007). In accordance with the geographic position, continental climatic features dominate. The distribution of soils is strongly influenced by bedrock and topography. The main soil types are vertisols, cambisols and fluvisols (European Soil Bureau Network 2005; Protic *et al.* 2005). The valleys of the Crni and Beli Timok follow graben structures (Jović 1997). In the east, the Crni Timok flows into the valley of the Veliki Timok, and in the west, it is connected to the valley of the Velika Morava.

As input data, we employ a DEM created from the contour lines digitized from topographic maps scaled 1:10,000 and interpolated using the GRASS GIS tool v.surf.rst (Mitasova *et al.* 2005). The settlements were identified through field surveys and can be dated from the Bronze Age to medieval times (Škundrić 2009, 2012).

**Fig. 7:**
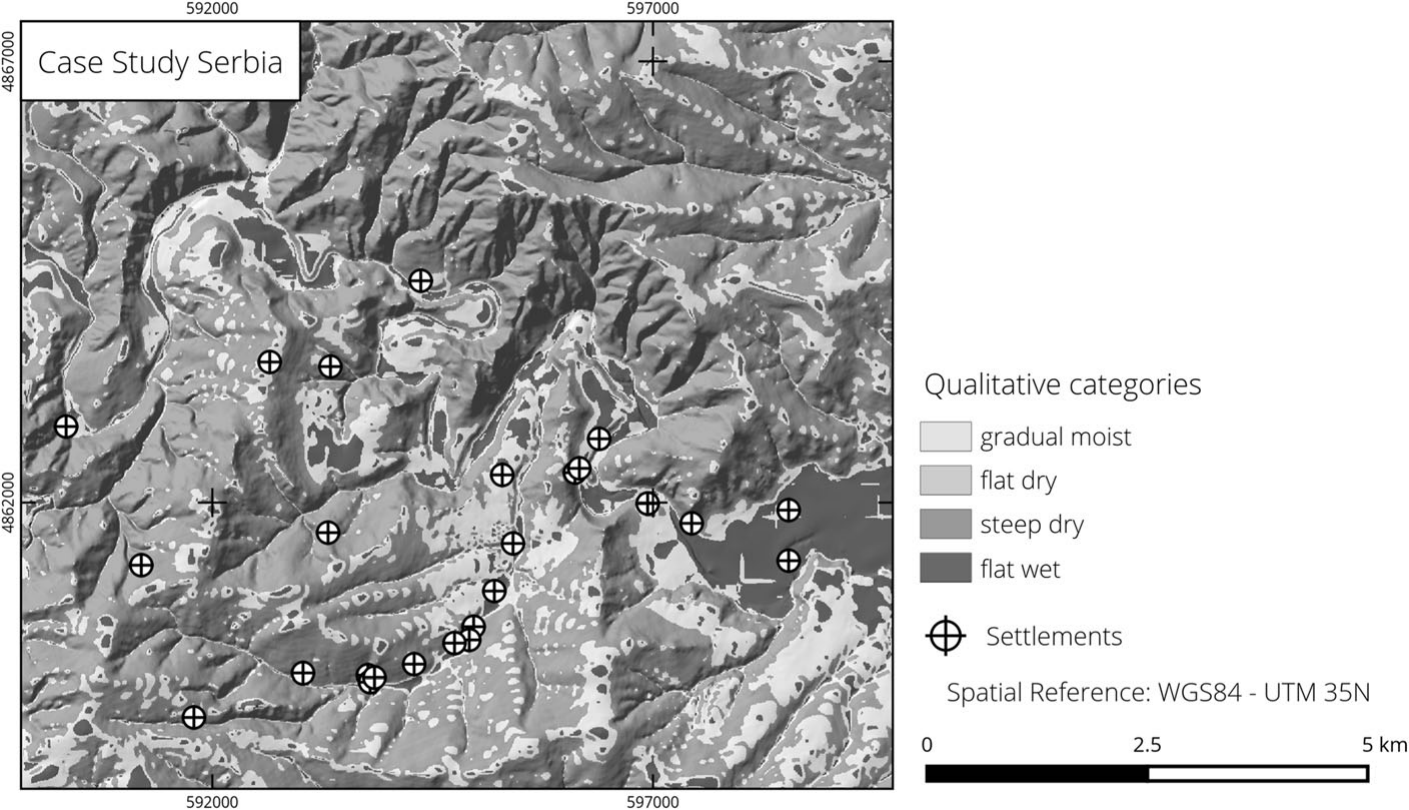
Study area in North-East Serbia (settlements from Škundrić 2012; elevation data derived from Kartirano u Vojnogeografskom Institutu 1968a, b, c, d)

The resulting maps (Figs. 6, middle and 7) exhibit problems in DEM creation using contour lines, particularly evident in the step-like character of the slopes (Hutchinson and Gallant 2000). With this in mind, our analysis included much of the research area in the category steep dry (Fig. 7). Footslopes, valley bottoms, hilltops and catchment divides are subsumed in the category gradual moist (Fig. 6, middle). The flat dry category is nearly absent, while only few areas are characterized as flat wet, mainly the eastern floodplain of the Crni Timok river valley. Most settlements are located in steep dry environments (Table 3), although the fuzzy membership degrees for this category at the site locations are often low and have a median value of just over 0.25 (Fig. 5, middle). Moreover, many of the sites from steep dry areas are found in close proximity to gradual moist environments, which contained 36 % of the settlements. No sites were placed in the flat dry category, and only a small number appeared in flat wet areas.

The third case study focuses on the environs of Aleppo, in modern Syria. The region is a flat to gently rolling sedimentary plain composed of carbonaceous rocks and tectonic domes (Wirth 1971, p. 378). The present climate is warm-temperate (Csa after Köppen and Geiger) with hot, dry summers and an annual mean temperature of 17.3 °C (Rösner 1995, p. 25). The only perennial stream in the area is the Nahr al-Quwayq (Qoueiq). It starts north of Aleppo, in a mountainous region, passes the city and drains into the steppe-swamp of al-Matā. Due to scarce surface water, the groundwater resources are very important for regional water supply. Valuable water sources only occur around the Nahr al-Quwayq and the eastern and western environs of Aleppo (Wolfart 1966, pp. 11–15; Wolfart *et al.* 1967, p. 267). The predominant soils are Vertic Inceptisols (Rösner 1995, p. 23; Strebel 1967, p. 273), which constitute the most fertile soils of modern Syria (van Liere 1963, p. 116; Wirth 1971, p. 171).

As in the first case study, we use a 90 by 90 m DEM based on SRTM data provided by Jarvis *et al.* (2008). The settlements date mainly to the Bronze Age and were collected during field surveys (Del Fabbro 2012).

Our analysis revealed that two qualitative categories dominate: gradual moist, spanning over much of the hilly areas, and flat wet, which is characteristic of the widespread flat parts (Figs. 6, right and 8). Only small areas, the steepest of the region, are categorized as steep dry. As in the Western Turkey case, the flat dry category is absent. More than two thirds of the settlements are concentrated in flat wet environments, while the remainder appear in gradual moist areas (Table 3). However, many of the latter sites are located adjacent to flat wet areas. The distribution of the fuzzy membership degrees for the four categories at the site locations reveals a preference for areas that are highly flat wet. In nearly three quarters of the cases, the value was over 0.5 and the median was 1 (Fig. 5, right).

**Fig. 8:**
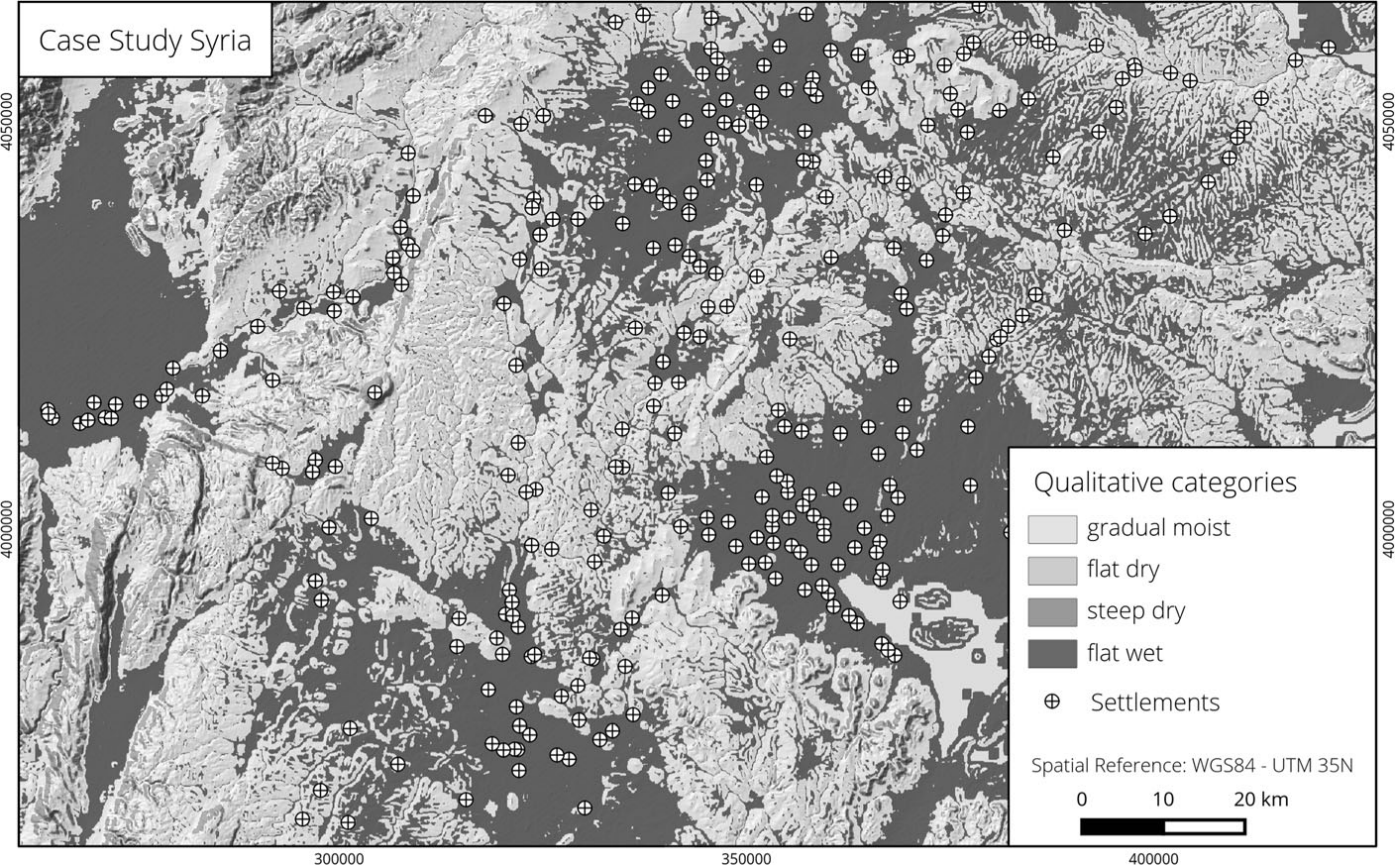
Study area in North-West Syria (settlements from Del Fabbro 2012; elevation data from Jarvis *et al.* 2008)

## Discussion

The Western Turkey case study produced clear results, revealing the effectiveness of the method in identifying the landscape preferences of prehistoric people. Seven out of ten settlements are found in gradual moist areas, while the rest are located close or on the border between steep dry and gradual moist environments. Despite the small data sample, there is little doubt that gradual moist environments were deliberately chosen for settlement, suggesting a strong correspondence between the qualitative categories that we have identified and the perception of prehistoric people when choosing the areas to inhabit. Gradual moist areas offer, relatively to the three other categories, a balance between surface inclination and water concentration. In the case of Western Turkey, this created the conditions that were thought to be most suitable for settlement. Given their high precision and accuracy, the results show predictive qualities (gain > 0.5) and thus serve as a good basis for future investigations.

In Eastern Serbia, the output is less clear, with no particular qualitative category preferred at first sight. One would assume that the best settlement locations are, just as in the Western Turkey case, the gradual moist areas; however, these contain only 36 % of all sites. The apparent mismatch, compared to the Western Turkey case, can have two possible explanations. Firstly, the already observed DEM generation issue and the particular shape of the terrain may have distorted the analysis procedure. The gradual moist membership degree map shows the close proximity of very high and very low values in situations where the geomorphologic characteristics barely change (Fig. 6, middle). This indicates the strong impact of the parameters that fix the threshold between the slope and MTI fuzzy classes in situations when one expects unpronounced variations in the distribution of the input variables. The higher resolution of the topographic data (10 m) allows for, and seemingly necessitates, a more detailed description of the terrain, which is not feasible using our chosen variables. Conversely, adding more detail would weigh the present surface features too much for them to be representative of a relief several thousand years old. Sudden membership degree variations would explain why the large majority of settlements, despite not being located in gradual moist environments, are situated in close vicinity to such areas. A second explanation is linked to the chronology of the settlements. Unlike the first case study, the settlements we used in Eastern Serbia spanned across a considerable amount of time. This entails large cultural and possibly some topographic changes. In light of such variation, it seems sensible to expect differences in how people perceived their surroundings and in the areas they chose to settle. Therefore, the fuzziness of the Eastern Serbia results reveals that, in order for the method to be effective, it is necessary for the geographical data to permit a good characterization of the relief and, at the same time, for the archaeological finds to be relatively contemporary.

The case study from the environs of Aleppo, in Syria, reveals the relative character of the employed qualitative categories. In this example, flat wet areas are greatly favoured over gradual moist ones. Furthermore, most of the settlements from gradual moist environments are located in close proximity to flat wet areas. To understand this particular preference, it is necessary to consider that the surroundings of Aleppo are characterized by an overall lack of water and that the four qualitative categories are defined in relative position to one another rather than universally. Hence, a gradual moist or flat wet area from Western Turkey does not have the same characteristics as a gradual moist or flat wet area near Aleppo. In the latter case, both areas are characterized by much smaller MTI values to account for the difference in water concentration.^5^ Nevertheless, in all situations, flat wet areas have the highest concentration of water. It thus seems reasonable that in an arid environment, such as the environs of Aleppo, people chose to settle in the areas with the most water. This preference is clearly visible in our results which indicate a predilection for locations that are highly flat wet (Fig. 5, right), even in areas of more pronounced relief (e.g. the top three sites in Fig. 6, right). Despite these observations, the high predisposition for settling in flat wet areas does not produce results with predictive quality, since the high precision is offset by low accuracy. Therefore, unlike the Western Turkey example, the gain is smaller than 0.5, as nearly half of the entire Aleppo region is included in the flat wet category.

The qualitative categories we identified probably resemble the way people categorized their landscape in all but the second case study. Humans were able to perceive and choose areas which correspond to gradual moist in Western Turkey and flat wet in Syria. Therefore, in these two contexts, the calculated qualitative categories represent landscape categories^6^ that people actively employed, together with other factors, when deciding which places to inhabit.

Numerous other elements participated both in the categorization of the landscape and especially in selecting a settlement’s location. Slope and MTI are good choices for understanding how people categorized their landscape, but it is obvious from the three case studies that the two elements alone can hardly produce an accurate picture. This emerges most clearly in the Syrian example, where the qualitative category preferred for settlement construction, flat wet, covers much of the study area. A refinement of the results would certainly be preferable with the help of additional information.

The near complete absence of flat dry areas from the three case studies reveals difficulties in identifying this particular qualitative category. This occurs, on one hand, because of poor SRTM resolution, which makes watersheds hard to delimit. On the other hand, this effect is an artefact of the MTI calculation, since areas with small slope usually correspond to high MTI values. Hence, uphill plateaus are erroneously categorized as flat wet instead of flat dry.

Finally, it is possible that some of the settlements located in floodplains, classified as flat wet, are covered today by sediments and have thus not found their place in our analysis (Bintliff 1992; Kraft *et al.* 1980; Stock *et al.* 2013). Floodplains were undoubtedly significant areas of the prehistoric and Mediterranean economy (Horden and Purcell 2000, pp. 186–190). Nevertheless, despite their important role, it was not necessary for people to settle actually in the floodplains in order to have access to them. The category gradual moist offers suitable conditions for permanent settlement and good access to these economically valuable locations, a situation well illustrated in our Western Turkey case study (Fig. 4, right). Future studies will have to reveal the degree to which floodplains were used as settlement space. In the meantime, we can only work with the available data.

## Conclusion

Using the method presented here, we have shown that the “cold” scientific measurements of different environment elements can be translated in terms of human perception. The divide between environment and landscape may be crossed by resorting to fuzzy logic and modelling the data on the basis of qualitative categories. While the full complexity of human-landscape interaction is far from being attained, facets of this process are certainly revealed.

Environment conditions and cultural factors affect the clarity of the results. In some areas, such as Western Turkey, the categorization is straightforward and the correspondence to archaeological data is extremely good. Similarly, in regions such as the surroundings of Aleppo, there is a clear preference for a particular landscape category. However, in this case, the variation in environment conditions is too small to be accurately characterized with the chosen variables alone; more factors need to be integrated to increase precision. At the same time, the example from Eastern Serbia suggests that the method may not be suited for particular terrain configurations. This case study also indicates the necessity for relative stability in terms of cultural factors, since people with different cultural backgrounds often categorized the landscape in a different manner.

Improvements can be added by considering different types of archaeological information, by varying the cultural conditions and by integrating more than two variables. The settlement data considered in this study can be doubled with information regarding the location of cemeteries or votive offerings, revealing more complex patterns of landscape use and environment perception. Furthermore, employing the method in a region containing evidence from groups sharing different cultural ideas, such as in Eastern Serbia, may serve to observe how cultural changes impacted peoples’ perception of the area. Finally, adding more variables would obviously make a more detailed categorization possible, although this would exponentially increase the complexity of the method and the time required to implement it.

## Footnotes

1 Of course the categories that people employed were likely also influenced by the knowledge that they accumulated from previous experiences, possibly coming from other contexts. However, unless the connection between different contexts and their influence on each other can be traced, it is difficult to incorporate such extra-contextual elements into the construction of the fuzzy classes.

2 An example of this is the slope variable employed in this paper.

3 The number of parameters is directly dependent on the number of fuzzy classes used to split the variables.

4 Although the use of a convex hull leads to simplifications in terms of territorial delimitation, we chose to employ this calculation because it produces results that can be easily reproduced.

5 This mimics the way the inhabitants of those lands would have categorised their landscapes, based solely on the information available to them in that particular environment.

6 Landscape categories can be considered to be a particular type of perceptive categories, as discussed in a forthcoming paper (Kuijpers et al. in preparation). Some ideas can also be encountered in a series or previous works (Hausmair 2015; Kuijpers 2014; Popa 2014).
